# Effectiveness of proprotein convertase subtilisin/kexin‐9 monoclonal antibody treatment on plasma lipoprotein(a) concentrations in patients with elevated lipoprotein(a) attending a clinic

**DOI:** 10.1002/clc.23607

**Published:** 2021-05-06

**Authors:** Anindita Chakraborty, Jing Pang, Dick C. Chan, Wendy Barnett, Ann Marie Woodward, Mary Vorster, Gerald F. Watts

**Affiliations:** ^1^ School of Medicine, Faculty of Health and Medical Sciences University of Western Australia Perth Australia; ^2^ Lipid Disorders Clinic, Cardiometabolic Services, Department of Cardiology Royal Perth Hospital Perth Australia

**Keywords:** atherosclerotic cardiovascular disease, LDL‐cholesterol, lipoprotein(a), proprotein convertase subtilisin/kexin‐9 monoclonal antibodies

## Abstract

**Background:**

Lipoprotein(a) (Lp[a]) is a causal risk factor for atherosclerotic cardiovascular disease (ASCVD). Proprotein convertase subtilisin/kexin‐9 monoclonal antibodies (PCSK9mAbs) can lower Lp(a) levels in clinical trials, but their effects in patients with elevated Lp(a) in clinical practice remain unclear.

**Aims:**

To investigate the effectiveness and safety of PCSK9mAbs in lowering plasma Lp(a) in patients with elevated Lp(a) concentrations in a lipid clinic.

**Methods:**

This was an open‐label study of 53 adult patients with elevated Lp(a) concentration (≥0.5 g/L). Clinical, biochemical, and safety data were collected before and on treatment with evolocumab or alirocumab over a mean period of 11 months.

**Results:**

Treatment with a PCSK9mAb resulted in a significant reduction of 0.29 g/L (−22%) in plasma Lp(a) concentration (p<.001). There were also significant reductions in low‐density lipoprotein‐cholesterol (LDL‐C) (−53%), remnant‐cholesterol (−12%) and apolipoprotein B (−43%) concentrations. The change in Lp(a) concentration was significantly different from a comparable group of 35 patients with elevated Lp(a) who were not treated with a PCSK9mAb (−22% vs. −2%, p<.001). The reduction in Lp(a) concentration was not associated with the corresponding changes in LDL‐C, remnant‐cholesterol, and apolipoprotein B (p>.05 in all). 7.5% and 47% of the patients attained a target concentration of Lp(a) <0.5 g/L and LDL‐C <1.8 mmol/L, respectively. PCSK9mAbs were well tolerated, the common adverse effects being pharyngitis (9.4%), nasal congestion (7.6%), myalgia (9.4%), diarrhoea (7.6%), arthralgia (9.4%) and injection site reactions (11%).

**Conclusion:**

PCSK9mAbs can effectively and safely lower plasma Lp(a) concentrations in patients with elevated Lp(a) in clinical practice; the impact of the fall in Lp(a) on ASCVD outcomes requires further investigation.

## 1 INTRODUCTION

Lipoprotein(a) (Lp[a]) is a low‐density lipoprotein (LDL)‐like particle with potent atherogenic, pro‐inflammatory, and anti‐thrombolytic properties.^1^, ^2^, ^3^, ^4^ Lp(a) comprises a molecule of apolipoprotein(a) covalently bound to apoB‐100 (apoB) via a disulphide bond.^5^ Elevated Lp(a) is the most common inherited disorder of lipoprotein metabolism, affecting approximately 20% of the general population.^1^, ^5^ Classical epidemiology and Mendelian randomization studies consistently demonstrate that elevated Lp(a) is an independent and causal risk factor for atherosclerotic cardiovascular disease (ASCVD), especially in women;^1^, ^2^, ^3^, ^4^, ^6^, ^7^ this also applies to high‐risk patients treated with statins.^8^, ^9^, ^10^ However, a lack of effective treatments for lowering Lp(a) remains a major challenge in the management of patients with elevated Lp(a).^2^, ^3^, ^4^, ^11^

Proprotein convertase subtilisin/kexin‐9 (PCSK9) is a key regulator of hepatic receptors involved in the metabolism of apoB‐containing lipoproteins.^12^ Inhibition of PCSK9 by a monoclonal antibody (mAb), such as evolocumab and alirocumab, can effectively reduce plasma concentrations of LDL‐cholesterol (LDL‐C) by up to 60%.^13^ Hence, PCSK9mAbs are used for the treatment of elevated LDL‐C in patients with familial hypercholesterolaemia (FH) and/or ASCVD on background maximum tolerated dose of statin, or in such patients with statin intolerance.^14^ Recent clinical trials and meta‐analyses also demonstrate that PCSK9mAbs can lower Lp(a) by up to 30% and this may partly account for the benefits of these agents in reducing ASCVD events.^15^, ^16^, ^17^, ^18^ However, current guidelines do not recommend the use of a PCSK9mAb to specifically lower elevated Lp(a).^2^, ^3^, ^4^, ^14^

While inhibition of PCSK9 is efficacious in lowering Lp(a) in clinical trials,^15^, ^16^, ^17^, ^18^ its effectiveness and safety have not yet been formally evaluated in real‐world clinical practice particularly in patients with elevated Lp(a).^19^, ^20^, ^21^ In the present study, we assessed the effectiveness and safety of PCSK9mAbs in lowering Lp(a) in patients with elevated Lp(a) attending a lipid clinic. We further investigated whether the change in plasma concentration of Lp(a) was associated with the corresponding changes in plasma LDL‐C and other lipids and lipoproteins.

## 2 METHODS

### 2.1 Selection of patients

Fifty‐three patients with elevated Lp(a) from the Lipid Disorders Clinics, Department of Cardiology, Royal Perth Hospital were included in the study. Patients were selected for having a plasma Lp(a) concentrations ≥0.5 g/L.^2^ Fifty patients were on evolocumab according to pharmaceutical benefits scheme (PBS) criteria and three patients were on alirocumab in a product familiarization program.^22^ These patients were selected from a large pool of clinic patients who were receiving treatment with these agents.

### 2.2 Study design

The study was a sequential, open‐label study of the effect of PCSK9mAbs in patients with elevated Lp(a), carried out over a period from 2015 to 2020. Forty‐seven patients were on 140 mg fortnightly (*n* = 44), monthly (*n* = 2) or six‐weekly (*n* = 1) and three on 420 mg monthly subcutaneous injections of evolocumab; two received 150 mg fortnightly and one 75 mg monthly injections of alirocumab. Patients attended regular clinic visits, where they were reviewed by a nurse specialist and/or a consultant physician and received best standard of care; clinical and biochemical data were collected and compared before and on treatment with evolocumab or alirocumab. For comparison, 35 patients with elevated Lp(a), attending the clinic and did not receive evolocumab or alirocumab, were also studied as a control group. At the time of recruitment, the 35 patients had plasma LDL‐cholesterol levels above guideline recommended target of <1.8 mmol/L but were not eligible for receiving a PCSK9mAb according to the PBS criteria in Australia. Clinical audit approval was granted by the Royal Perth Hospital (Quality Activity 29009).

### 2.3 Clinical data

The following clinical data were recorded: Age, gender, ethnicity, body mass index (BMI), blood pressure; personal history of coronary artery disease (CAD), cerebrovascular event (a stroke or transient ischemic attack), peripheral artery disease (PAD), hypertension, diabetes, or kidney disease; family history of ASCVD; current medications. CAD was defined as a history of either; (i) myocardial infarction, (ii) angioplasty, or (iii) coronary artery bypass grafting.^23^ Hypertension was defined as systolic and diastolic blood pressure >140 or >90 mmHg, respectively or on anti‐hypertensive medication. Diabetes was defined as a fasting glucose concentration of ≥7.0 mmol/L, random glucose of ≥11.1 mmol/L, glycated hemoglobin >6.5% or use of anti‐diabetic medication (insulin or oral hypoglycaemic agents), and obesity as BMI ≥30 kg/m^2^. Renal function was determined by the estimated glomerular filtration rate (eGFR) using the modification of diet in renal disease (MDRD) equation. Similar information to the above was collected in the 35 clinic patients with elevated Lp(a) who were not treated with PCSK9mAb.

### 2.4 Diagnosis of FH

FH was defined according to the Dutch lipid clinic network (DLCN) criteria and/or the presence of a pathogenic mutation in the *LDLR*, *APOB*, and *PCSK9* genes.^23^ Genetic testing was done by the Cardiovascular Genetics Laboratory, PathWest Laboratory Medicine, Royal Perth Hospital, as previously described.^23^

### 2.5 Definition of statin intolerance

Statin intolerance was defined as the inability (complete or partial) to tolerate at least two different statins (one statin at the lowest starting daily dose and another statin at any daily dose) due to clinically important adverse effects, such as musculoskeletal symptoms and markedly elevated plasma creatinine kinase and/or hepatic aminotransferase.^24^

### 2.6 Definition of treatment adverse events

Adverse events (AEs) during PCSK9mAb therapy were defined as general systemic symptoms and injection site reactions. General systemic symptoms were flu‐like (pharyngitis, nasal congestion), musculoskeletal (myalgia, back pain, arthralgia), gastrointestinal (diarrhea, constipation, nausea, abdominal discomfort/pain), and other (headache and fatigue). Injection site reactions were defined as a cluster of erythema, pain, bruising, swelling, induration, rash, or pruritus around the injection sites. The severity of the above was assessed by the numerical rating scale on a 0–10 scale, any score of >4 defined as the presence of a clinically significant AE.^25^

### 2.7 Clinical review of PCSK9mAb therapy

Patients first attended a nurse‐managed clinic for education and training on the administration, dosage, and frequency of the subcutaneous injections, as well as on the storage requirements of the injectate and potential AEs. Typically, follow‐up reviews were done via telephone, or with intermittent clinic visits as required. Patients were telephoned by the specialist nurse every fortnightly or monthly, based on the injection schedules to elicit and discuss clinical progress and AEs. The first post‐treatment fasting blood test for lipids, lipoproteins, and other biochemical analytes were carried out 6 weeks after the first injection, followed by blood tests at 3–4 monthly intervals; blood tests were not done during acute illness.

### 2.8 Biochemical analyses

Plasma glucose, liver (alanine transferase, gamma‐glutamyl transferase) and muscle (creatine kinase) enzymes, total cholesterol (TC), triglyceride and high‐density lipoprotein‐cholesterol (HDL‐C) concentrations were determined by standard enzymatic methods (Architect c16000 Analyzer, Abbott Diagnostics, Abbott Laboratories, Abbott Park, IL). LDL‐C was calculated by the Friedewald equation or measured by a direct assay with plasma triglycerides >4.5 mmol/L. Non‐HDL‐C was calculated by subtracting HDL‐C from TC, whereas remnant‐cholesterol (REM‐C) was calculated by subtracting HDL‐C and LDL‐C from TC. LDL‐C was adjusted for Lp(a)‐cholesterol by subtracting 30% of the individual's Lp(a) total mass from the plasma LDL‐cholesterol concentration.^26^ Total apoB and Lp(a) (Quantia Lp(a) assay and standards) were determined by immunoassay (Abbott Laboratories, Abbott Park, IL). Lp(a) measurement using this method has been significantly correlated with a liquid chromatography‐mass spectrometry method.^27^ Interassay coefficient of variation of all measurements were <6%.

### 2.9 Statistical analyses

Statistical analyses were carried out using the SPSS Statistics (Version 25; Armonk, New York; IBM Corp). Data were presented as mean ± SD unless otherwise specified. The Shapiro–Wilk test was used to determine whether variables were normally distributed. Skewed variables, including plasma triglyceride, REM‐C, and Lp(a) concentrations were log‐transformed. Gender differences in plasma levels of pre‐treatment and post‐treatment Lp(a) were compared using independent *t*‐test. Treatment effects were analyzed using paired t‐tests. Change in plasma Lp(a) concentration between the PCSK9mAb treated and the non‐PCSK9 treated group were compared using general linear modeling with baseline as a covariate. Absolute changes in Lp(a) levels with PCSK9mAb treatment according to quartiles for Lp(a) were compared using ANOVA with multiple comparisons. The proportion of patients attaining LDL‐C and Lp(a) targets after PCSK9mAb treatment were calculated with reference to recommendations in current guidelines.^2^, ^14^ Associations between on‐treatment changes in Lp(a) concentrations and other variables were assessed by simple and multiple linear regression. Statistical significance was defined at the 5% level.

## 3 RESULTS

### 3.1 Patient characteristics

Table 1 shows the clinical and biochemical characteristics of the patients studied. They were mostly late middle‐aged, Caucasians and overweight, with normal blood pressure, glucose, and renal function. The majority of the patients had a family history of CAD; half had a history of CAD and one‐fifth a history of cerebral event or PAD. Eighty‐three percent of the patients had phenotypically probable/definite FH. Of the 36 patients who underwent genetic testing, 64% had an FH mutation, most commonly in the *LDLR* gene (*n* = 20). More than half the patients were current or ex‐smokers and hypertensive, 13% had diabetes, 42% were obese and 38% were statin intolerant. Approximately one‐third of the patients were on statin monotherapy, ezetimibe monotherapy or the combination of a statin and ezetimibe; 15% of patients were on a mean dose of 65 mg/day atorvastatin, 20% were on 28 mg/day rosuvastatin, 8% were on 65 mg/day simvastatin, and 2% were on 20 mg/day pravastatin. There were no statistically significant differences in age, gender, BMI, blood pressures, plasma lipid and lipoproteins concentration (p > .05 for all) between the intervention group and the control group of 35 clinic patients with elevated Lp(a) who were not on PCSK9mAb (Supplementary Table 1).

**TABLE 1:** Demographic, clinical and biochemical characteristics of the 53 patients studied

Age (years)	59.7 ± 10.5
Male, *n* (%)	24 (45.3)
Ethnicity	
Caucasian, *n* (%)	50 (94.3)
Asian, *n* (%)	1 (1.9)
Others (mixed/aboriginal/African/middle Eastern), *n* (%)	2 (3.8)
Body mass index (kg/m^2^)	28.8 ± 5.1
Systolic blood pressure (mmHg)	131 ± 12
Diastolic blood pressure (mmHg)	78 ± 10
Glucose (mmol/L)	5.7 ± 1.7
HbA1c (%)	5.8 ± 0.6
Serum creatinine (μmol/L)	78.8 ± 23.3
Estimated glomerular filtration rate (ml/min/1.73 m^2^)	80.7 ± 14.8
Family history of coronary artery disease, n (%)	49 (92.5)
Personal history of coronary artery disease, n (%)	29 (54.7)
Personal history of cerebral or peripheral artery disease, n (%)	10 (18.9)
DLCN criteria score^a^ >5, n (%)	44 (83.0)
Smokers (current/ex), n (%)	29 (54.7)
Hypertension, n (%)	30 (56.6)
Type 2 Diabetes, n (%)	7 (13.2)
Obesity, n (%)	22 (41.5)
Statin intolerant, n (%)	20 (37.7)
On lipid‐lowering medication, *n* (%)	49 (92.5)
Statin, *n* (%)	33 (62.3)
Statin monotherapy, *n* (%)	15 (28.3)
Ezetimibe monotherapy, *n* (%)	16 (30.2)
Statin in combination with ezetimibe, *n* (%)	18 (34.0)
Atorvastatin, *n* (%)	8 (15.1)
Dose (mg/day)	65 ± 20.7
Rosuvastatin, *n* (%)	20 (37.7)
Dose (mg/day)	28.3 ± 15.2
Simvastatin, *n* (%)	4 (7.5)
Dose (mg/day)	65 ± 30
Pravastatin, n (%)	1 (1.9)
Dose (mg/day)	20
Fibrates, *n* (%)	1 (1.9)
Fish oils, *n* (%)	5 (9.4)

*Note*: Values represented as mean ± SD or number (%). Genetic test for familial hypercholesterolaemia (FH) was carried out in 36 patients: 23 (63.9%) had an FH mutation: 20 in the *LDLR* gene; two in the *APOB* gene and one in the *PCSK9* gene.

Abbreviation: LDL‐C, low‐density lipoprotein‐cholesterol.

^a^ Dutch lipid clinic network (DLCN) criteria score refers to the score without the genetic analysis component.

### 3.2 Effect of PCSK9mAb therapy

**FIGURE 1:**
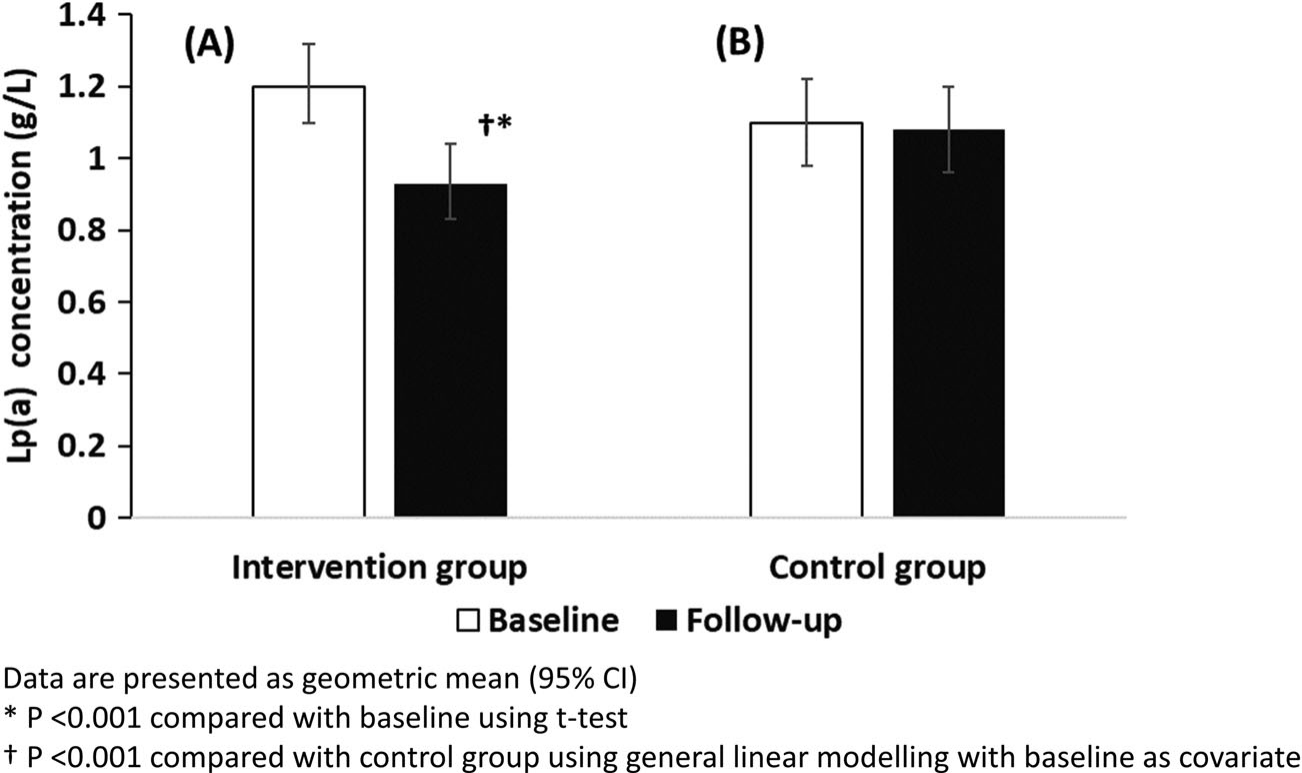
Plasma Lp(a) concentrations at baseline and at follow‐up in the PCSK9mAb intervention group (A) and in a clinic group not treated (control) with a PCSK9mAb (B)

Table 2 shows plasma lipid, lipoprotein and apolipoprotein concentrations of the 53 patients with elevated Lp(a) before and during therapy with a PCSK9mAb: this intervention significantly lowered the plasma concentration of TC (−36%, p < .001), triglyceride (−9%, p < .005), non‐HDL‐C (−46%, p < .001), LDL‐C (−53%, p < .001), adjusted LDL‐C (−58%, p < .001), REM‐C (−12%, p < .05), apoB (−43%, p < .001) and Lp(a) (−22%, p < .001) (see Supplementary Figure 1). There were no statistically significant differences between men and women in the pre‐treatment and post‐treatment Lp(a) levels (1.17 vs. 1.23 g/L, and 0.90 vs. 0.95 g/L, respectively; p > .05 in both), as well as the absolute change in plasma Lp(a) levels with a PCSK9mAb (−0.31% vs. −0.28%; p = .788). The fall in Lp(a) was significantly different (−22% vs. −2%, p < .001) from the change in Lp(a) in the 35 clinic patients not treated with PCSK9mAb therapy (see Figure 1).

**TABLE 2:** Plasma lipid, lipoprotein, apolipoprotein and Lp(a) concentrations pre‐ and on‐treatment with a PCSK9mAb

Lipid and lipoprotein variables	Pre‐treatment values	On‐treatment values	Absolute change in values	p‐value
TC, mmol/L	6.61 ± 0.27	4.14 ± 0.18	− 2.47 ± 0.17	<.001
TG, mmol/L^a^	1.78 (1.52, 2.08)	1.51 (1.30, 1.76)	− 0.34 (− 0.59, − 0.08)	.002
HDL‐C, mmol/L	1.34 ± 0.05	1.35 ± 0.04	0.01 ± 0.03	.853
Non‐HDL‐C, mmol/L	5.27 ± 0.27	2.79 ± 0.18	− 2.48 ± 0.17	<.001
LDL‐C, mmol/L	4.33 ± 0.22	2.03 ± 0.15	− 2.30 ± 0.15	<.001
LDL‐C adjusted, mmol/L^b^	3.32 ± 0.24	1.25 ± 0.16	− 2.08 ± 0.15	<.001
REM‐C, mmol/L^a^	0.82 (0.71–0.95)	0.67 (0.58–0.77)	− 0.17 (− 0.29, − 0.06)	<.001
Apo B, g/L	1.38 ± 0.06	0.78 ± 0.04	− 0.60 ± 0.04	<.001
Lp(a), g/L^a^	1.20 (1.08–1.34)	0.93 (0.83–1.04)	− 0.29 (− 0.35, − 0.24)	<.001

*Note*: Values represented as mean ± SEM or geometric mean and (95% confidence intervals).

Abbreviations: ApoB, apolipoprotein‐B; HDL‐C, high density lipoprotein‐cholesterol; LDL‐C: low‐density lipoprotein‐cholesterol; PCSK9mAb, proprotein convertase subtilisin/kexin‐9 monoclonal antibodies; REM‐C, remnant‐cholesterol; TC, total cholesterol; TG, triglycerides; Lp(a), lipoprotein(a).

^a^ Skewed variables with log transformation.

^b^ Modification of LDL‐cholesterol to account for the 30% of cholesterol contained within the Lp(a) particles.

When stratifying patients into quartiles on the basis of pre‐treatment Lp(a) levels (Table 3), PCSK9mAb treatment resulted in a significant absolute reduction of plasma Lp(a) levels in patients from each quartile (p < .001 for all). Moreover, there was a significant trend to an increase in absolute reductions of plasma Lp(a) levels in patients from the lowest quartile to the highest quartile (i.e., −0.15, −0.23, −0.31, and −0.50 g/L, respectively; ANOVA p < .001). Patients with pre‐treatment Lp(a) concentration in the highest (fourth) quartile had a greater reduction of Lp(a) than those in the first, second, and third quartiles (p < .05 for all). In contrast, we found that the percentage reductions of plasma Lp(a) were comparable among quartiles (from −20.7% to −23.9%).

**TABLE 3:** Plasma lipoprotein(a) concentrations pre‐ and on‐treatment with a PCSK9mAb according to quartiles of pre‐treatment lipoprotein(a) levels

Quartiles (g/L)	Pre‐treatment (g/L)	On‐treatment (g/L)	Absolute change (g/L)	Percentage change (%)
Quartile 1 (0.56–0.89)	0.75 (0.68, 0.82)	0.59 (0.50, 0.69)*	−0.15 (−0.19, −0.11)***	−20.7 (−27.6, −13.8)
Quartile 2 (0.90–1.13)	1.03 (0.99, 1.08)	0.80 (0.73, 0.88)*	−0.23 (−0.31, −0.15)***	−21.5 (−28.9, −14.2)
Quartile 3 (1.15–1.66)	1.40 (1.30, 1.51)	1.09 (0.96, 1.23)*	−0.31 (−0.40, −0.21)**	−21.9 (−28.7, −15.1)
Quartile 4 (1.67–3.03)	2.04 (1.82, 2.27)	1.54 (1.34, 1.76)*	−0.50 (−0.63, −0.36)	−23.9 (−30.1, −17.6)

*Note*: Values represented as geometric mean and (95% confidence intervals).

Abbreviation: PCSK9mAb, proprotein convertase subtilisin/kexin‐9 monoclonal antibodies.

^*^ p < .001 compared to pre‐treatment Lp(a) levels within each quartile;

^**^ p < .05 compared to Quartile 4 for absolute Lp(a) reduction.

^***^ p < .001 compared to Quartile 4 for absolute Lp(a) reduction.

**FIGURE 2:**
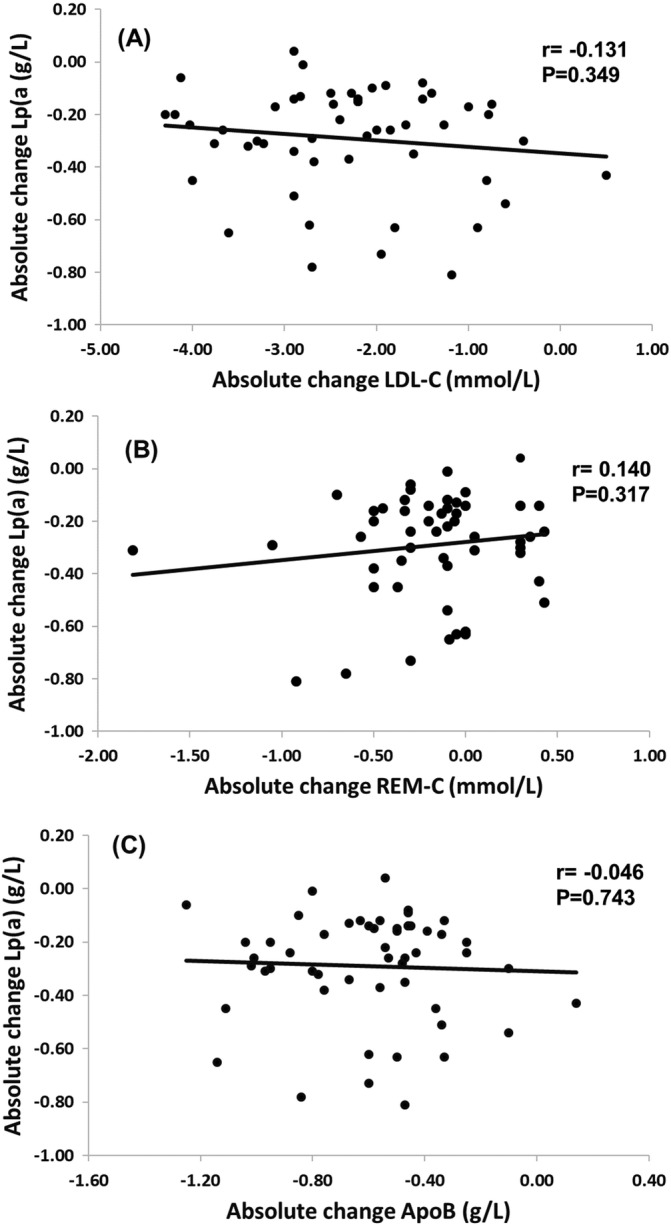
Associations between the absolute changes in plasma Lp(a) concentration with PCSK9mAb treatment and corresponding changes in plasma concentrations of low‐density lipoprotein‐cholesterol (LDL‐C) (A), REM‐C (B) and apoB (C), respectively

The absolute change in Lp(a) concentration was not significantly correlated (p > .05 in all) with the corresponding changes in LDL‐C, REM‐C and apoB concentrations (Figure 2). Likewise, the percentage change in Lp(a) concentration was also not significantly correlated with the corresponding changes in LDL‐C, REM‐C and apoB concentrations (data not shown). There was no significant difference in the reduction of Lp(a) concentration between patients with and without an FH mutation (−24% vs. −21%, p > .05) nor between patients with and without background statin treatment (−23% vs. −21%, p > .05). 7.5% and 47% of the patients attained a target concentration of Lp(a) <0.5 g/L and LDL‐cholesterol <1.8 mmol/L, respectively.^2^, ^14^

### 3.3 Regression analysis

In univariate regression, pre‐treatment Lp(a) concentration was significantly and inversely associated with the absolute reduction, but not with percentage reduction, of Lp(a) in response to PCSK9mAb treatment (r = −0.707, p < .001). In a multiple linear regression model including age, gender, BMI, baseline LDL‐cholesterol concentration and treatment duration, only the baseline Lp(a) concentration was independently and significantly correlated with the absolute reduction in Lp(a) concentration (standardized B‐coefficient −0.597, p < .001).

### 3.4 Adverse effects of PCSK9mAb therapy

The common adverse effects reported by the patients treated with PCSK9mAbs are shown in Supplementary Table 2. Twenty‐one patients (40%) reported at least one AE. The most common AEs reported by the patients were pharyngitis (9%), nasal congestion (8%), myalgia (9%), diarrhea (8%), arthralgia (9%); injection‐site reactions were reported by 11% of the patients. Treatment was generally well‐tolerated, except for one patient who developed severe nasopharyngitis after 24 months and had to discontinue treatment with evolocumab. There were no significant within‐group differences in plasma glucose, HbA1c, plasma liver and muscle enzymes, and plasma creatinine concentrations in relation to treatment with evolocumab or alirocumab (data not shown).

## 4 DISCUSSION

### 4.1 Main findings

Our principal finding was that in patients with elevated Lp(a) attending a lipid clinic, PCSK9 inhibition with a mAb resulted in a significant reduction in Lp(a) concentration and was well tolerated over a mean period of 11 months. We also demonstrate that the reduction in Lp(a) with PCSK9mAb was significantly and inversely correlated with pre‐treatment Lp(a) concentration, but not with the changes in LDL‐cholesterol, remnant‐cholesterol or apoB concentration.

### 4.2 Previous studies

Few studies have reported the real‐world effectiveness of PCSKmAb on Lp(a) concentration in a clinical setting.^19^, ^20^ In a 2‐centre study from the UK, Kohli et al found that short‐term PCSK9 inhibitor therapy (6–12 weeks) significantly lowered plasma Lp(a) concentration by 28% in high‐risk patients, the majority of whom had heterozygous FH.^19^ In a prospective clinical cohort of high‐risk patients (almost half of them had ACSVD and one‐third were FH), Kaufman et al showed a 23% reduction in Lp(a) concentration after 1 year of PCSK9mAb treatment in lipid clinic patients.^20^ However, none of these studies specifically examined the effect of PCSK9mAb in patients selected for having elevated Lp(a) concentration. The characteristics of our patients were comparable with those in previous reports and were consistent with the reimbursement criteria for PCSK9mAbs in such patients not attaining LDL‐C targets.^19^, ^20^, ^21^ We have extended previous studies by investigating the effect of PCSKmAb on plasma Lp(a) concentration in patients with high Lp(a).

### 4.3 Lp(a) reduction

Several large clinical trials of PCSK9mAb treatment have consistently demonstrated a significant reduction in Lp(a) concentration.^15^, ^16^, ^17^, ^18^ In the ODYSSEY OUTCOMES trial,^18^, ^28^ alirocumab reduced Lp(a) by −0.20 g/L (−22%) in a subset of patients with elevated Lp(a). In an observational study of four high‐risk patients with elevated Lp(a), 6 month PCSK9mAb treatment lowered plasma Lp(a) levels by 12%.^29^ We also reported that in statin‐treated patients with elevated Lp(a), PCSK9mAb treatment resulted in an absolute reduction of plasma Lp(a) concentration by −0.23 g/L (−23%), which is mediated by increased catabolism of Lp(a) particles.^30^ This is consistent with the notion that PCSK9 inhibition could enhance Lp(a) removal by upregulating hepatic LDL receptor activity.^12^, ^30^, ^31^ Previous data also suggest that PCSK9 could directly bind to Lp(a) particles in the circulation.^32^, ^33^ Hence, another possible mechanism of action of PCSK9mAb treatment is the formation of a circulating triple immune complex of PCSK9mAb with PCSK9‐bound Lp(a), followed by removal of this complex from the circulation. However, it may only represent a very small percentage of total Lp(a) that is complexed. Despite clinical trials usually having very restricted inclusion and exclusion criteria, the magnitude of Lp(a) reduction with PCSK9mAb treatment in the present real‐world study was comparable to those reported in clinical trial settings.^15^, ^16^, ^17^, ^18^, ^30^ Moreover, we also found a significant trend to an increase in absolute reductions of plasma Lp(a) levels in patients from the lowest quartile to the highest quartile (Table 3). This is consistent with our correlational analysis that the absolute reduction, but not relative reduction, in Lp(a) with PCSK9mAb treatment was directly dependent on the pre‐treatment Lp(a) concentration. This observation could be due to a regression‐to‐the‐mean effect, or to individuals with higher baseline Lp(a) concentration having a truly greater absolute Lp(a) reduction, the lack of association with proportional reduction in Lp(a) being expected on mathematical grounds.^34^

### 4.4 Association with LDL‐cholesterol, remnant‐cholesterol and apoB

PCSK9 is a key regulator of LDL and other hepatic receptor involved in the metabolism of apoB‐100 containing lipoproteins.^12^ However, we found that the reduction of Lp(a) was not significantly associated with the fall in LDL‐C with PCSK9mAb treatment, consistent with previous studies showing a discordant association between the reductions in Lp(a) and LDL‐C with PCSK9mAb treatment.^35^, ^36^, ^37^, ^38^ This implies that the LDLR may not play a major role in the catabolism of Lp(a) particles. However, the lack of association between the reductions in Lp(a) and LDL‐cholesterol may be due to the limited number of patients in the study. Hence, our inferences need to be interpreted with caution and confirmed in a study with a larger sample size. Previous experimental data suggest that Lp(a) can be preferably catabolized via non‐LDLR pathways, such as VLDL receptor, LDLR‐related protein 1, megalin/gp330, scavenger receptor class B type 1, and plasminogen receptors.^39^

### 4.5 Safety and adverse effects

Recent randomized controlled trials (RCTs) and meta‐analyses have favorable safety and good tolerability profile of PCSK9mAbs.^13^, ^16^, ^17^, ^18^, ^19^, ^28^, ^30^, ^34^, ^35^ These findings may not be fully applicable to clinical practice which encompasses patients with diverse co‐morbidities and multiple therapies. There are also limited data on the safety of PCSK9mAb in real‐world settings.^19^, ^20^, ^21^, ^40^ In the present study, the most common reported adverse effects were nasopharyngitis, diarrhea, myalgia, arthralgia, as well as injection‐site reactions. These findings are compatible with data from clinical trials.^13^, ^17^, ^18^, ^28^, ^30^ Although 40% of patients reported at least one AE, the severity of those AEs was insufficient to stop treatment.

### 4.6 Study strengths and limitations

Our sample size was relatively small, but the findings were comparable to previous studies in real‐world settings of PCSK9mAb.^9^, ^19^, ^20^ We also focused on patients with elevated Lp(a). We did not employ a formal placebo‐controlled design to study the effects of PCSK9mAb treatment on plasma Lp(a) concentrations. However, we demonstrated that the significant effect on Lp(a) lowering remained significant compared with Lp(a) variations in 35 clinic patients who did not receive PCSK9mAb treatment. Hence, our findings are unlikely to be confounded by a regression‐to‐the‐mean effect. The background use of statins may have a differential impact on the effect of PCSK9mAb on plasma Lp(a) concentration.^41^ However, we found that the effects of PCSK9mAb on Lp(a) concentration was independent of background statin therapy. The Quantia Lp(a) assay used in our study is not fully isoform independent, but we have found a good agreement with a liquid chromatography‐mass spectrometry method.^27^ Duration of the follow‐up period of 11 months may not be sufficient to fully assess the long‐term safety profile of PCSK9mAb treatment. We did not specifically assess the effect of PCSK9mAb treatment on neurocognitive effects. However, previous clinical trials have not shown an increased risk of adverse neurocognitive effects with PCSK9mAb therapy.^13^, ^42^

### 4.7 Clinical implications

Several clinical practice guidelines recommend measuring plasma Lp(a) concentration in patients with high‐risk of ASCVD.^2^, ^3^, ^4^, ^14^ However, current guidelines do not specify the degree to which Lp(a) needs to be lowered to prevent ASCVD events. The recent FOURIER and ODYSSEY Outcomes trials demonstrate that the reduction in Lp(a) could partly mediate the benefit of PCSK9mAb treatment in reducing ASCVD risk in high‐risk patients, particularly in those with elevated Lp(a).^17^, ^18^, ^28^ In the ODYSSEY trial, each 5 mg/dl reduction in Lp(a) with alirocumab was associated with a 2.5% relative reduction in total ASCVD event.^28^ This is generally in agreement with a population study showing that a reduction of Lp(a) concentration by 50 mg/dl lowered ASCVD risk by 20% in a secondary prevention setting.^43^ The effect of Lp(a) on the risk of ASCVD events appears to be proportional to the absolute reduction in Lp(a), not the percent reduction.^28^ The mean reduction in Lp(a) of 0.29 g/L with a PCSK9mAb could be approximately translated into 12%–15% reduction of ASCVD.^28^, ^43^ However, this speculation remains to be demonstrated.

Patients with elevated Lp(a) who also have CAD and FH, as in our sample population, remain at high residual risk of ASCVD despite statin treatment.^2^, ^3^, ^4^, ^8^ Recent studies suggest that lowering Lp(a) by approximately 1 g/L could reduce major ASCVD events by 22%.^44^ As demonstrated in the present study, PCSK9mAbs cannot achieve this degree of lowering of Lp(a) concentrations. Attaining such an effective reduction in Lp(a) requires the use of RNA‐based therapies that target the apo(a) gene transcripts.^45^

## 5 CONCLUSION

In clinic patients with elevated Lp(a), most of whom had CAD and FH, PCSK9mAb treatment safely and significantly lowered the plasma concentration of Lp(a), with a greater reduction in LDL‐C and apoB. Whether the incremental reduction in Lp(a) translates into a decrease in ASCVD events remains unclear. The aggregate fall in atherogenic apoB‐containing lipoproteins, including Lp(a), with PCSK9mAb, is likely due to increased hepatic clearance of these lipoproteins^46^, ^47^ and probably mediates the principal cardiovascular benefits of this treatment.^18^, ^19^, ^28^, ^48^, ^49^

## CONFLICT OF INTEREST

Gerald F. Watts has received honoraria for lectures and advisory boards or research grants from Amgen Inc., Arrowhead, AstraZeneca, Esperion, Kowa, Novartis, Regeneron, and Sanofi. Anindita Chakraborty, Jing Pang, Dick C. Chan, Wendy Barnett, Ann Marie Woodward, and Mary Vorster have no disclosures.

## Supporting information


**Appendix S1**: Supporting Information
