# Supplementary material for: Effectiveness of proprotein convertase subtilisin/kexin‐9 monoclonal antibody treatment on plasma lipoprotein(a) concentrations in patients with elevated lipoprotein(a) attending a clinic
Source: Clin Cardiol. 2021 May 6;44(6):805–13. doi: 10.1002/clc.23607 (PMC8207967; doi:10.1002/clc.23607)
Supplement: Supplementary file 1 — Appendix S1: Supporting Information [file CLC-44-805-s001.pdf]

Supplementary tables

Supplementary TABLE 1 Demographic, clinical and biochemical characteristics of the 35 clinic patients not treated with a PCSK9mAb

Age (years)	57.2 ± 8.5
Male, n (%)	14 (40)
Body mass index (kg/m^2)	29.4 ± 5.2
Systolic blood pressure (mmHg)	126 ± 18
Diastolic blood pressure (mmHg)	80 ± 9.3
Personal history of coronary artery disease, n (%)	6 (35.0)
On lipid-lowering medication, n (%)	12 (34.3)
Statin, n (%)	12 (34.3)
Statin monotherapy, n (%)	10 (28.6)
Statin in combination with ezetimibe, n (%)	2 (5.7)
Atorvastatin, n (%)	3 (8.6)
Dose (mg/day)	53.3 ± 23.1
Rosuvastatin, n (%)	8 (22.9)
Dose (mg/day)	8.3 ± 6.1
Pravastatin, n (%)	1 (2.9)
Dose (mg/day)	20
TC, mmol/L	7.26 ± 1.05
TG, mmol/L*	1.92 (1.62-2.28)
HDL-C, mmol/L	1.44 ± 0.33
Non-HDL-C, mmol/L	5.82 ± 0.99
LDL-C, mmol/L	4.81 ± 0.91
REM-C, mmol/L*	0.89 (0.75-1.06)
ApoB, g/L	1.41± 0.27
Lp(a), g/L*	1.10 (0.97-1.25)

Values represented as mean ± SD, geometric mean (95% confidence intervals) or number (%)

*Skewed variables with log transformation

TC: Total cholesterol, TG: triglycerides, HDL-C: High density lipoprotein- cholesterol, LDL-C: low-density lipoprotein-cholesterol, REM-C: Remnant-cholesterol, ApoB: Apolipoprotein-B and Lp(a): Lipoprotein(a)

Genetic test for familial hypercholesterolaemia (FH) was carried out in 23 patients: 2 (8.7%) had an FH mutation.

Supplementary TABLE 2 Adverse events reported by patients following treatment with a PCSK9mAb

**Adverse events**	**Number (%)**
Pharyngitis	5 (9.4%)
Nasal Congestion	4 (7.6%)
Myalgia	5 (9.4%)
Headache	2 (3.8%)
Back pain	2 (3.8%)
Diarrhoea	4 (7.6%)
Constipation	1 (1.9%)
Nausea	2 (3.8%)
Abdominal discomfort	3 (5.7%)
Fatigue	4 (7.6%)
Arthralgia	5 (9.4%)
Injection site reactions*	6 (11.3%)

*Injection site reactions include erythema, pain bruising swelling, induration, rash and pruritus

Supplementary Figure 1 Mean percentage changes in lipid, lipoprotein, apoB and Lp(a) concentrations on treatment with a PCSK9mAb

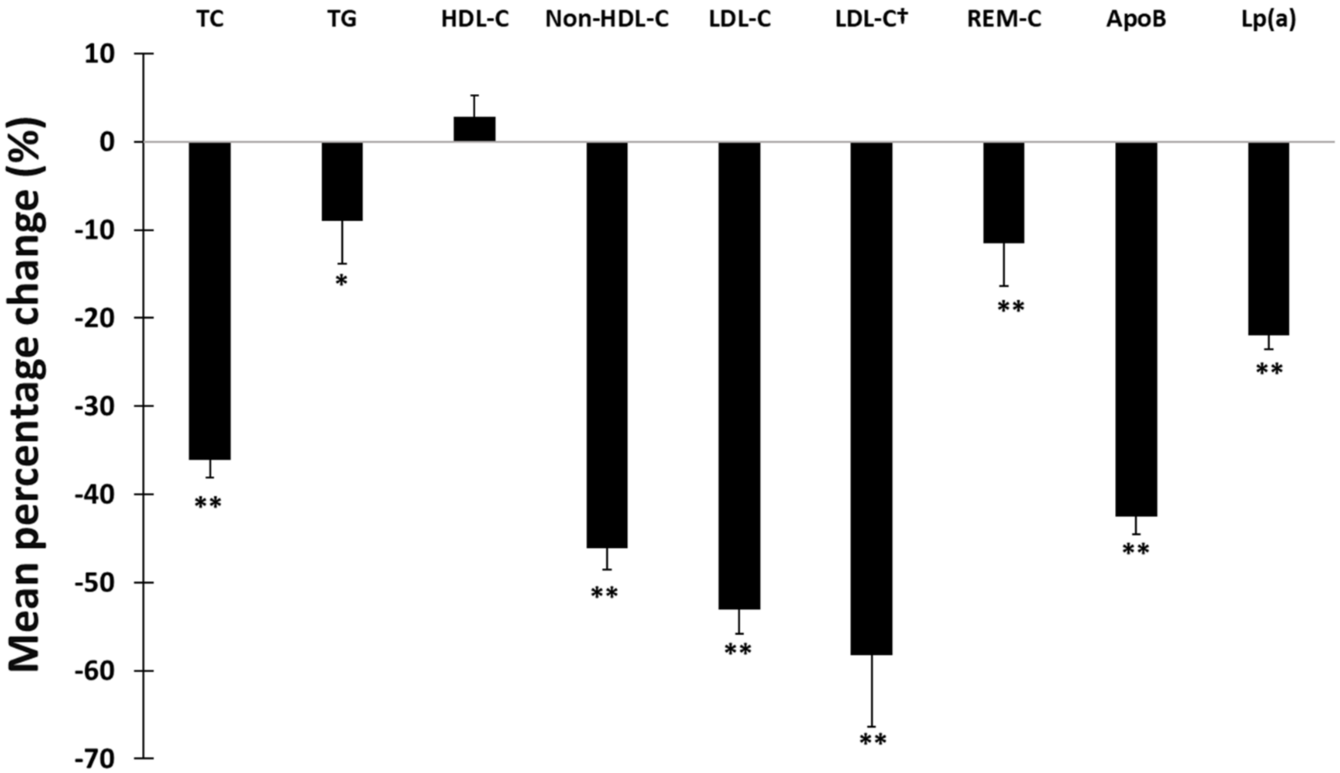

Data are presented as a mean percentage change (SEM)
TC: Total cholesterol, TG: triglycerides, HDL-C: High density lipoprotein- cholesterol, LDL-C: low-density lipoprotein- cholesterol, REM-C: Remnant-cholesterol, ApoB: Apolipoprotein-B and Lp(a): Lipoprotein(a)
†Adjustment of LDL-C to account for the 30% of cholesterol contained within the Lp(a) particles
*P< 0.005; ** P<0.001
